# Multisteroid LC–MS/MS assay for glucocorticoids and androgens and its application in Addison's disease

**DOI:** 10.1530/EC-13-0023

**Published:** 2013-07-04

**Authors:** Paal Methlie, Steinar Hustad, Ralf Kellman, Bjørg Almås, Martina M Erichsen, Eystein S Husebye, Kristian Løvås

**Affiliations:** 1 Department of Clinical ScienceUniversity of BergenN-5021, BergenNorway; 2 The Hormone LaboratoryHaukeland University HospitalN-5021, BergenNorway; 3 Department of MedicineHaukeland University HospitalBergen, N-5021Norway

**Keywords:** liquid chromatography mass spectrometry, androgens, glucocorticoids, adrenal insufficiency, Addison's disease

## Abstract

**Objective:**

Liquid chromatography–tandem mass spectrometry (LC–MS/MS) offers superior analytical specificity compared with immunoassays, but it is not available in many regions and hospitals due to expensive instrumentation and tedious sample preparation. Thus, we developed an automated, high-throughput LC–MS/MS assay for simultaneous quantification of ten endogenous and synthetic steroids targeting diseases of the hypothalamic–pituitary–adrenal axis and gonads.

**Methods:**

Deuterated internal standards were added to 85 μl serum and processed by liquid–liquid extraction. Cortisol, cortisone, prednisolone, prednisone, 11-deoxycortisol, dexamethasone, testosterone, androstenedione and progesterone were resolved by ultra-high-pressure chromatography on a reversed-phase column in 6.1 min and detected by triple-quadrupole mass spectrometry. The method was used to assess steroid profiles in women with Addison's disease (AD, *n*=156) and blood donors (BDs, *n*=102).

**Results:**

Precisions ranged from 4.5 to 10.1% relative standard deviations (RSD), accuracies from 95 to 108% and extraction recoveries from 60 to 84%. The method was practically free of matrix effects and robust to individual differences in serum composition. Most postmenopausal AD women had extremely low androstenedione concentrations, below 0.14 nmol/l, and median testosterone concentrations of 0.15 nmol/l (interquartile range 0.00–0.41), considerably lower than those of postmenopausal BDs (1.28 nmol/l (0.96–1.64) and 0.65 nmol/l (0.56–1.10) respectively). AD women in fertile years had androstenedione concentrations of 1.18 nmol/l (0.71–1.76) and testosterone concentrations of 0.44 nmol/l (0.22–0.63), approximately half of those found in BDs of corresponding age.

**Conclusion:**

This LC–MS/MS assay provides highly sensitive and specific assessments of glucocorticoids and androgens with low sample volumes and is suitable for endocrine laboratories and research. Its utility has been demonstrated in a large cohort of women with AD, and the data suggest that women with AD are particularly androgen deficient after menopause.

## Introduction

Glucocorticoid and androgen hormone measurements play a decisive role in the diagnosis and management of many disorders of the hypothalamic–pituitary–adrenal (HPA) axis and gonads. Examples include Addison's disease (AD), Cushing's syndrome (CS) and congenital adrenal hyperplasia, male hypogonadism and female hyperandrogenism. Although immunoassays have largely been used to measure steroid hormones, over the last decade, mass spectrometry (MS) has increasingly been adopted as it offers superior analytical specificity and accuracy. However, MS is not commonly available to clinicians in many regions and hospitals. The high cost of MS instruments, lack of expertise, and cumbersome and time-consuming sample preparation procedures are factors likely to slow its adoption. The development of simple, reliable, and cost- and labour-efficient methods is, therefore, important.

Liquid chromatography coupled to tandem mass spectrometry (LC–MS/MS) is a highly selective mode of detection. It enables simultaneous determination of multiple steroids at very low concentrations in a single analytical run. Although many papers have been published on LC–MS/MS assays that measure one or a few steroid hormones, only few papers have reported methods that extensively exploit the capability of multiplex determination of steroids in human serum (1, 2, 3). This strategy could simplify the laboratory set-up by eliminating the need to use numerous separate methods and make instrument utilization more efficient.

In MS/MS, the specificity is increased by collision-induced fragmentation of the analyte into a molecular fingerprint that could be identified by monitoring two or more fragments. However, many previously published multisteroid LC–MS/MS assays monitor only one mass transition (collision fragment) per analyte (2, 3, 4, 5, 6) and consequently have an increased risk of not detecting isobaric interference. Another analytical concern is that inter-individual differences in serum composition, such as lipids and binding proteins, could potentially impact measurements. This is often not investigated at all. Most published methods also require relatively large sample volumes to provide sufficient sensitivity (1, 2, 4, 7, 8) and have long run times (1, 2, 4, 7).

The work-up and management of endocrine disorders commonly involve the administration of synthetic steroid hormones. The determination of synthetic glucocorticoids used in dynamic endocrine testing and pharmacological treatment may thus be a valuable supplement to the measurement of endogenous steroids. For example, the measurements of serum dexamethasone (DXM) could provide pharmacokinetic data useful in the evaluation of the DXM suppression test (9, 10), and therapeutic drug monitoring of prednisone and prednisolone has the potential to optimize treatment (11, 12). Because of the widespread use of these glucocorticoids and impact on endogenous cortisol levels, information on serum levels may also be valuable in the setting of a routine endocrine laboratory.

Therefore, the objective of this study was to develop a LC–MS/MS method that quantifies endogenous and synthetic glucocorticoid hormones as well as endogenous androgens. With a sample volume of only 85 μl serum, the method provides a highly sensitive, fast, comprehensive and cost-effective evaluation of patients with a range of disorders related to glucocorticoid and androgen hormones. Its utility was demonstrated by comparing the steroid profiles of women with AD with those of healthy female blood donors (BDs). To our knowledge, this report is the first to investigate androgen levels in a large cohort of women with AD using highly specific MS.

## Materials and methods

### Chemicals

Cortisol, cortisone, prednisolone, prednisone, 11-deoxycortisol (11DOC), testosterone, androstenedione, 17α-hydroxyprogesterone (17OHP) and progesterone were obtained from Steraloids, Inc. (Newport, RI, USA), and DXM was obtained from Alfa Aesar (Ward Hill, MA, USA).

The deuterated internal standards (ISs) cortisol-d4 (cortisol-9,11,12,12-d4, 97–98% atom D), cortisone-d2 (4-pregnen-17α,21-diol-3,11,20-trione-1,2-d2, >98% atom D), prednisolone-d6 (1,4-pregnadien-11β,17α,21-triol-3,20-dione-2,4,6,6,21,21-d6, >98% atom D), DXM-d4 (DXM-4,6α,21,21-d4, 96–98% atom D), 11DOC-d2 (4-pregnen-17α,21-diol-3,20-dione-21,21-d2, >96% atom D), testosterone-d3 (testosterone-16,16,17-d3, >98% atom D), androstenedione-d7 (4-androsten-3,17-dione-2,2,4,6,6,16,16-d7, >98% atom D), 17OHP-d8 (4-pregnen-17α-ol-3,20-dione-2,2,4,6,6,21,21,21-d8, >98% atom D) and progesterone-d9 (progesterone-2-2-4-6-6-17α,21,21,21-d9, >98% atom D) were purchased from CDN Isotopes (Pointe-Claire, Quebec, Canada).

MS-grade acetonitrile (ACN), methanol (MeOH), formic acid (>98%) and ammonium formate (>98%) were obtained from Merck. Milli-Q water purification system (Millipore, Burlington, MA, USA) was used to prepare de-ionized water (>18 MΩcm). Dextran-coated charcoal (product number C6241) was obtained from Sigma–Aldrich.

### Calibrators, ISs and quality controls

Steroid-free human serum was prepared with activated dextran-coated charcoal according to the manufacturer's protocol. Serum was obtained from healthy BDs.

All steroid hormones were separately dissolved in MeOH at concentrations of 3 mM for the analytes and 500 μM for the ISs. Two separate mixtures of the analytes and the ISs, designated standard substock and IS substock, were prepared in MeOH. The standard substock contained each steroid hormone at a concentration 100 times that of the highest working calibrator. Working calibrators were prepared by serially diluting the standard substock 1:4 in MeOH and then by adding 2 ml of each dilution to 198 ml steroid-free serum. The final working calibrators covered the following measuring ranges: 1.95–2000 nmol/l, cortisol; 0.98–250 nmol/l, cortisone; 0.98–1000 nmol/l, prednisolone; 0.98–1000 nmol/l, prednisone; 0.06–250 nmol/l, DXM; 0.10–25 nmol/l, 11DOC; 0.02–75.0 nmol/l, testosterone; 0.12–125 nmol/l, androstenedione; 0.24–250 nmol/l, 17OHP; 0.24–250 nmol/l, progesterone; and a blank control. Six calibrators were used for all the analytes, except for cortisone and 11DOC (five calibrators) and testosterone and DXM (seven calibrators).

The concentrations of each of the ISs in the IS substock were 100 times that of the working IS solution. The working IS solution was prepared by diluting the IS substock with H_2_O:MeOH (1:1). The final working IS solution contained 850 nmol/l cortisol-d4, 850 nmol/l cortisone-d2, 425 nmol/l prednisolone-d6, 106 nmol/l DXM-d4, 128 nmol/l 11DOC-d2, 51.0 nmol/l androstenedione-d7, 31.9 nmol/l testosterone-d3, 106 nmol/l 17OHP-d8 and 106 nmol/l progesterone-d9.

Quality controls (QCs) were prepared in steroid-free serum at three levels (low, medium and high) by adding each steroid hormone to a final concentration corresponding to 0.30, 5.0 and 50% of the highest working standard. All standards, calibrators and QCs were stored in Nunc cryovials (Nalge Nunc International, Roskilde, Denmark) at −80 °C.

### Sample preparation procedure

To 85 μl of serum, working calibrators or QCs, 10 μl of an IS were added, mixed and equilibrated for 1 h. The steroid hormones were then extracted with 825 μl ethyl acetate:hexane (80:20). The supernatant (600 μl) was transferred to a new vial and washed with 50 μl ammonium formate buffer (pH 9.0, 0.1 M). Subsequently, 500 μl of organic phase were transferred to a new vial and evaporated at 50 °C for 25 min under N_2_ flow and finally reconstituted in 50 μl H_2_O:formic acid:MeOH (49.9:0.1:50). Sample preparation was automated on a Hamilton Star (Hamilton Robotics, Inc., Reno, NV, USA) using exclusively 1.1 ml glass vials. Mixing steps were performed by repeated pipette aspiration/dispense cycles. Centrifugation was not necessary because the aquatic and organic phases separated within minutes.

### LC–MS/MS conditions

An Agilent 1290 UPLC system (Santa Clara, CA, USA) equipped with a thermostated autosampler (4 °C) and a degasser was used for chromatographic separation. Processed serum (5 μl) was separated over a Zorbax RRHD C18 reversed-phase column (50×2.1 mm, 1.8 μm particle size, Agilent) maintained at 30 °C. The mobile phases were water (A) and ACN (B) with 0.1% formic acid. The column was developed with stepwise linear gradient elution according to the following timetable: 0.00 min, 15% B; 0.25 min, 15% B; 0.50 min, 25% B; 1.70 min, 28.5% B; 3.80 min, 40% B; 4.20 min, 50% B; 4.90 min, 70% B; 5.00 min, 95% B; 5.50 min, 95% B; 5.60 min, 15% B; and 6.10 min, 15% B. Flow rate was 1000 μl/min and the column effluent was delivered to the mass spectrometer in the time window 1.1–5.0 min.

The LC system was coupled to an Applied Biosystems/MDS SCIEX API 5500 triple-quadrupole mass spectrometer operating with electrospray ionization (Applied Biosystems/MDS, Foster City, CA, USA). Manual tuning and selection of mass transitions were performed using a T-split to mix the 5 μl/min infusion containing 500 nmol/l of the compound with the 1000 μl/min mobile phase flow (35% B). Using this set-up, additional Q1 and Q3 scans (90–500 *m/z*) were performed by varying the ion source parameters (voltage, temperature and gas flows) to investigate the stability of the substances under conservative and extreme conditions, as well as the formation of adducts. Ion source parameters and mass transitions are reported in Table 1. Entrance potential was 10 V, collision gas was set to medium and resolution was set to unit for Q1 and Q3. To increase scan time for each analyte, the multiple reaction monitoring (MRM) was divided into seven periods based on the time of elution. All hardware was managed using the Analyst Software (version 1.5.1; Applied Biosystems/MDS).

### Quantification and QC

Quantification was based on peak area ratios of the analyte to the corresponding IS. Standard curves were computed using a linear least-squares regression analysis with 1/x or 1/x^2^ weighting to assign priority to the lower range of the calibration curves.

To accept analytical runs, the back-calculated concentrations of at least 80% of the working calibrators had to be within ±15% (20% for the lowest calibrator) of their nominal concentration. Two sets of QCs (low, medium and high) were run in each batch (96 samples). The measured concentrations of at least one QC at each level and at least 75% of all the QCs were required to be within ±15% their nominal levels. Interference was suspected if the peak area ratio of quantifier to qualifier transitions deviated more than ±40% from the corresponding ratio of the third highest calibrator.

### Method validation

Within-day and between-day precisions were investigated at three levels by running the QCs in replicates (*n*=6) on 12 different days. The coefficients of variance (CV) were calculated using one-way ANOVA. Accuracy (%) was computed as (measured concentration/nominal concentration)×100, using the means of all the measured concentrations. The method was also compared with immunoassays using the Passing–Bablok regression and Bland–Altman plots.

The limits of detection (LoDs) and lower limits of quantification (LLoQs) were determined by adding the analytes at progressively lower levels to steroid-free serum and analysing samples in replicates (*n*=6). The LoD was defined as the concentration with a signal-to-noise ratio above 3 and the LLoQ as the lowest concentration with a CV below 20% and accuracy within ±20%.

Recovery, matrix effects (MEs) and linearity were investigated in charcoal-stripped serum obtained from different individuals, adopting the principles suggested by Matuszewski *et al*. (13). Analytes were added to steroid-free serum obtained from six individuals; aliquots of each serum were spiked to six levels corresponding to the working standards. Duplicate sera were spiked both before extraction (pre-extract spike) and after evaporation (post-extract spike) by adding 20 μl of the standard substock per 980 μl serum. Moreover, analytes were added to a ‘blank’ reconstitution solvent (reference). Reference samples were analysed immediately before, in the middle and after the pre/post-spike series. Extraction recoveries (ERs), ion-source-related MEs and overall process efficiency (PE) were calculated from the mean of duplicate samples according to the formulas given in Supplementary Table 1, see section on supplementary data given at the end of this article. Evaporation of the organic phase during sample preparation was closely monitored by gravimetric measurements of simultaneously run control samples, and ERs, MEs and PE were adjusted accordingly.

Linearity was evaluated based on residual plots and correlation coefficients. Relative MEs (14), that is, the impact of inter-individual differences in serum composition, were assessed by computing the slope constant based on each of the six pre-extraction spiked sera from the recovery experiment. The slope constants were derived from weighted (1/x or 1/x^2^) least-squares linear regression of nominal concentration vs analyte:IS peak area ratio.

All analytes and ISs and other potentially interfering steroid hormones (Supplementary Table 2, see section on supplementary data given at the end of this article) were prepared separately in water at concentrations of 2000 nmol/l. These included all drugs containing steroid hormones that are commercially available in Norway. We also reviewed data from random samples received at the Hormone Laboratory (*n*=926), from rheumatic patients on prednisolone therapy (*n*=50) and from patients taking DXM (*n*=25) for interfering peaks and deviations of the quantifier:qualifier peak area ratio.

Stability was investigated by computing the mean of triplet analysis of QCs at three concentration levels after storage under different conditions. Stability was examined after storage at ambient temperature for 1, 2, 4, 8, 24 and 96 h, in a refrigerator (4 °C) for 1, 2, 5, 10, 20 and 30 days, and in a freezer (−20 °C) for 1, 2 and 3 months. Freeze–thaw stability was determined after five cycles of freezing to −80 °C. The stability of the processed samples (*n*=96) in the autosampler was studied by repeatedly analysing the same batch on 4 consecutive days.

### Application: steroid profiles of women with AD and BDs

The method was applied to samples obtained from the Norwegian registry of organ-specific autoimmune diseases. The study population has been described previously (15), and sera obtained from women with AD (*n*=156) and female BDs (*n*=102) were analysed. The Regional Committee for Medical Ethics of Western Norway and the National Data Inspectorate approved the study. All of the patients gave written informed consent after complete explanation of the purpose and nature of all the procedures used. Most of the AD patients were on cortisone acetate replacement therapy, but five were using prednisolone. Ten patients had premature ovarian failure and seven were on DHEA replacement therapy. Blood samples were drawn between 0800 and 1600 h. For statistical analyses, the subjects were stratified into three groups: young (<40 years), middle aged (40–60 years) and old (>60 years). The groups were compared by the Kruskal–Wallis test using the Wilcoxon test as a *post hoc* analysis, and Bonferroni-adjusted *P* values are reported. *P*<0.05 was considered statistically significant.

## Results

### Precision, accuracy, lower limit of detection and quantification

**Figure 1:**
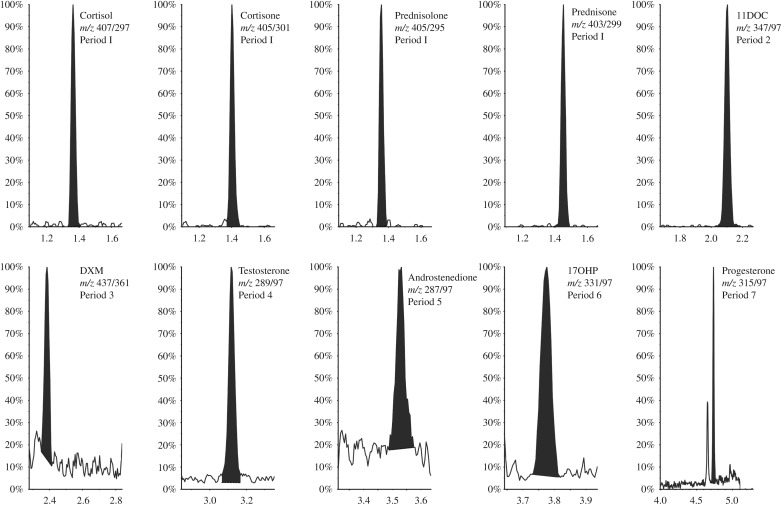
MS chromatograms of the quantifier mass transition of the lowest working calibrator for each analyte. The nominal concentrations were as follows: 1.95 nmol/l, cortisol; 0.98 nmol/l, cortisone; 0.98 nmol/l, prednisolone; 0.98 nmol/l, prednisone; 0.10 nmol/l, 11-deoxycortisol (11DOC); 0.06 nmol/l, dexamethasone (DXM); 0.02 nmol/l, testosterone; 0.12 nmol/l, androstenedione; 0.24 nmol/l, 17α-OH-progesterone (17OHP); and 0.24 nmol/l; progesterone. *m/z*, mass-to-charge ratio of the ionized compound and fragment.

**Figure 2:**
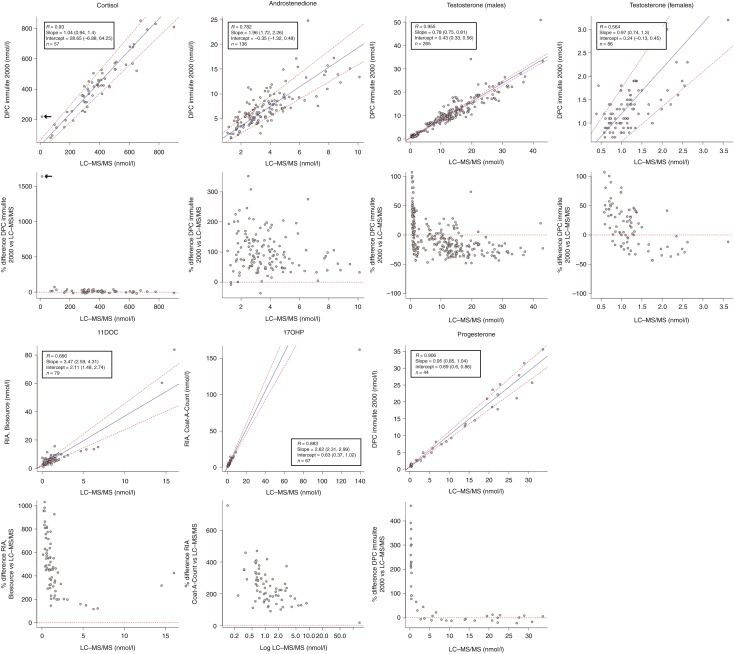
Comparison of the LC–MS/MS method with immunoassays available at the Hormone Laboratory. Statistical analyses were carried out using the Passing–Bablok regression (upper) and Bland–Altman plots (lower). Arrows in cortisol plots denote a patient put on prednisolone therapy in whom the immunoassay reported falsely elevated cortisol levels (220 vs 12 nmol/l).

Data from the precision and accuracy experiments are reported in Table 2. Total CV were ≤10.1% for all the compounds, and accuracies were in the range of 95–108%. The LoDs and LLoQs are reported in Table 3, and chromatograms of the lowest calibrators are shown in Fig. 1. Comparison of the LC–MS/MS method with immunoassays available in our laboratory is shown in Fig. 2.

### Recovery, linearity and MEs

Recovery and linearity were investigated in sera obtained from six individuals (Supplementary Table 1). The ERs and PE were high and consistent across low-to-high concentration levels. The ER was 60% for progesterone, and it ranged from 71 to 85% for the other analytes. There was no significant ion depression or enhancement. The method was linear for all the compounds, except for cortisone and 11DOC. For these two compounds, the highest calibrators of 250 and 25 nmol/l respectively were slightly underestimated, but had acceptable back-calculated accuracies above 90%.

The variance of the slope constants between different individuals provides a useful index of relative MEs. The CV of the slope constants were ≤3% for all the analytes (Supplementary Table 3, see section on supplementary data given at the end of this article). According to Matuszewski (14), this indicates that the method is robust to intra-individual differences in the serum. On running routine samples, it was observed that the peak areas of the ISs were consistent over a batch of 96 samples with CV typically in the range of 10–20%.

### Selectivity

Potential analytical interference of 48 synthetic and naturally occurring steroid hormones and metabolites (Supplementary Table 2) was investigated. All the analytes were chromatographically separated, except prednisone and prednisolone. Individual injection of these two compounds at 2000 nmol/l showed that the selected fragment ions of these glucocorticoids did not interfere in the negative ionization mode. In the systematic evaluation of chromatograms from 1000 routine analyses, suspicion of interference was very rare. The quantifier:qualifier peak area ratio was within ±40% of the expected value for all the analytes of all the samples.

### Stability

Steroid hormones in the serum were stable when stored at 4 °C or a lower temperature. After storage for up to 25 days at 4 °C or over 6 months at −20 °C, all the compounds were still measured within ±10% of their nominal levels with no apparent trends. The compounds were robust to at least five freeze–thaw cycles. Cortisone concentrations were within ±10% of the nominal levels after 48 h of storage at ambient temperature; however, for samples containing levels of 50 nmol/, concentrations declined to 86% after 96 h. Similarly, at this time point, the concentrations of samples with prednisone at an initial level of 50 nmol/l had declined to 84%. Reconstituted samples were stable for at least 4 days at 4 °C.

### Application: steroid profiles of women with AD and BDs

The steroid profiles of patients with AD and BDs are reported in Table 4. The method provided information on the cortisol and cortisone levels of patients on oral hydrocortisone replacement therapy and successfully identified the five patients taking prednisolone (prednisolone 317 nmol/l, range 220–419; prednisone 47.8 nmol/l, range 17.2–86). Nine AD patients had low levels of 11DOC (median 0.20; range 0.14–0.32 nmol/l).

Testosterone and androstenedione concentrations were above the LLoQs in all the BDs and most of the women with adrenal insufficiency. The two AD groups of women aged above 60 years and women with premature ovarian failure stood out as in them androgen levels were often not even detectable. Generally, patients with AD exhibited decreasing serum testosterone concentrations with age, and levels were significantly lower in the old group vs the young group (*P*=0.0195). This was in contrast to the testosterone levels in BDs, where no difference between the old and young groups was observed (*P*=1). The middle-aged BDs, however, had lower levels than the young group (*P*=0.003).

Serum androstenedione concentrations also declined with age in women with AD. Androstenedione concentrations in the old group were lower than those in the middle-aged group (*P*<0.00001), which again were lower than those of the young group (*P*=0.006). For BDs, androstenedione concentrations were not significantly different in the middle-aged group and the old group (*P*=0.160). The young group had higher levels relative to the middle-aged group (*P*=0.0002) and the old group (*P*=0.00007).

On comparing women with AD with BDs, it was found that testosterone levels were significantly lower in all the age-stratified groups (all *P*≤0.00020). Similarly, androstenedione concentrations were lowest in AD patients (all *P*≤0.00005). The levels of 17OHP in the old groups were lower in AD patients than in the BDs (*P*<0.00001). There were no systematic differences in serum cortisol and cortisone levels between AD patients and BDs, as expected since the timing of specimen collection was not standardized.

## Discussion

We developed a multiplexed LC–MS/MS assay that targets the work-up of adrenal and gonadal disorders. As demonstrated by the steroid profiles of women with AD, it allows the quantification of very low levels of androgens, as well as measurements of endogenous and synthetic glucocorticoids. The method is automated, requires only 85 μl serum and has a chromatographic run time of 6.1 min, which makes it suitable for use in routine endocrine laboratories as well as in research.

The panel of analytes includes the most commonly systemically used synthetic glucocorticoids. This could be particularly useful in a clinical setting as it enables a comprehensive assessment of the patient. For example, the overnight low-dose DXM suppression test is commonly used in the work-up of CS, but its diagnostic specificity of only 80% is a major drawback (16). The determination of serum DXM could help in identifying patients with false-positive tests due to reduced gastrointestinal DXM absorption or abnormal DXM metabolism. This strategy is indeed suggested by the Endocrine Society (17), but rarely implemented. Moreover, the detection of synthetic glucocorticoid hormones could reveal iatrogenic causes of CS as well as prevent unnecessary investigations in patients with iatrogenic cortisol suppression, both circumstances commonly encountered in the routine laboratory. Moreover, prednisolone and prednisone are commonly used as immunosuppressants and in the treatment of cancer. It is a clinical observation that these drugs show considerable inter-individual variation in dose required for therapeutic effect. Analogously, some patients are more resistant to adverse effects. Therapeutic monitoring of prednisolone and prednisone levels may ultimately optimize clinical response and reduce adverse effects, such as osteoporosis and cardiovascular disease (18, 19, 20).

Our LC–MS/MS assay allows the quantification of serum testosterone in the picomolar range found in women and children, which traditional immunoassays cannot measure reliably (21). We achieved a LLoQ for testosterone of less than one-fifth of that reported by Guo *et al*. (2) and Ceglarek *et al*. (3), and functional sensitivity was similar to that of methods employing labour-intensive derivatization procedures to enhance the ionization of androgens (5, 8). The LLoQs in the range of 0.02–1.97 nmol/l are, for most of the analytes, lower than those of previously published LC–MS/MS methods, although our method requires less serum volume (1, 2, 3, 4, 5, 7, 8). Combined with simultaneous determination of multiple hormones, low serum volume requirement is an advantage when the specimen is scarce, such as samples from children and population-based biobanks. The low sample volume used also facilitates high-throughput analysis by allowing unattended automation of the extraction procedure in a standard 96-well format using a liquid-handling robot. Within an 8-h workday, 288 samples can be prepared, and more than 200 samples per day can be analysed.

When hormones with different chemical properties are assayed simultaneously, there is a balance between obtaining high recoveries for all the compounds and eliminating substances that cause ion suppression or impact method ruggedness. Overall, extraction with ethyl acetate:hexane yielded high recoveries without significant ion suppression or enhancement. The clean extracts contributed to the long lifetime of the analytical column, which typically withstood thousands of injections.

Although MS is a highly specific analytical detector, it is necessary to chromatographically separate the steroid analytes from isobaric isomers and other interfering compounds. We found that the Zorbax RRHD C18 column performed well for both glucocorticoids and androgens. A reversed-phase column with phenyl-bonded stationary phase may better separate cortisol, cortisone, prednisolone and prednisone (22), but is incapable of resolving androgens from interfering compounds. Because the glucocorticoids formed specific adducts with formic acid in the negative ionization mode, the co-elution of prednisone and prednisolone was without significant cross-interference.

The increasing sensitivity of the latest generation of MS instruments has increased awareness and concern for isobaric interferences, in particular, in the picomolar range. Monitoring two mass transitions (i.e. collision fragments) adds another layer of specificity as an unexpected deviation of the ratio of the quantifier to qualifier peak areas should raise suspicion of interference (23, 24). Although monitoring two mass transitions reduces scan time and thus sensitivity and LLoQ, this strategy has been recommended (25) or advised as mandatory (26) for steroid analysis. Our assay monitors two mass transitions, which we believe is an important improvement compared with other multisteroid methods (2, 3, 4, 5, 6).

Inter-individual differences in serum composition could potentially impact measurements. For example, the ER of steroids in the serum may vary with the amount of binding proteins and lipids. An isotopic analogue of the analyte is commonly used as an IS to compensate for such loss. Nevertheless, it is still possible that the binding of the IS to various components in the serum is not in perfect equilibrium with the analyte. Another concern, particularly relevant to the increased resolution offered by ultra-high-pressure LC, is that the ionization efficiency of the analyte and IS may differ as they do not always co-elute exactly (27). Therefore, a strength of our method is the comprehensive validation of relative MEs, which often are not investigated at all (1, 2). The consistent results obtained when calibration curves were prepared for sera collected from different individuals indicate that the assay is robust to inter-individual differences in serum composition.

The clinical usefulness of the method was demonstrated by comparing steroid profiles of women with AD with those of BDs. The measurements of endogenous and synthetic glucocorticoids indicate the potential of the method to assess patients on glucocorticoid therapy. The five patients on prednisolone replacement therapy were identified by the detection and quantification of prednisone and prednisolone.

Herein, we report data on androgens in women with AD. To our knowledge, this study is the first to investigate testosterone and androstenedione concentrations in a large cohort of women with adrenal insufficiency using highly specific MS. To differentiate between premenopausal and postmenopausal status, we stratified the study subjects into young (<40 years), middle-aged (40–60 years) and old (>60 years) groups. The middle-aged group is expected to comprise a mixture of fertile and postmenopausal women. We found that in AD women aged below 40 years, androstenedione and testosterone concentrations were roughly 50% of those found in BDs. This is in line with studies indicating that androgens originate from the adrenal glands and ovaries at about equal ratios in fertile healthy females (28). An interesting finding is the very low androgen levels in women with AD aged above 60 years, as opposed to the levels in healthy females of comparable age. This may suggest that postmenopausal women with AD, in particular, are deficient of androgens. It also raises the question as to why the postmenopausal ovary in AD ceases to secrete androgens. Notably, there has long been a controversy whether the postmenopausal ovary indeed is a major androgen-producing organ (29, 30). A reasonable interpretation of our data could be that the premenopausal ovary is capable of *de novo* androgen synthesis, while the postmenopausal ovary depends on adrenal DHEA/S as the substrate to produce testosterone and androstenedione. This would explain the low androgen levels found in postmenopausal women with AD. Moreover, this may also explain some of the discrepant results of DHEA replacement in AD (15, 31, 32, 33) because the study populations investigated differ in gender, menopausal status and autoimmune ovarian failure. Conceivably, DHEA replacement therapy may be more effective in the postmenopausal AD women without autoimmune ovarian damage.

Alteration in binding globulin, for example, due to the use of oral contraceptives, could potentially influence the unbound levels of androgen in the serum. We did not measure SHBG levels, which is a limitation of our study. If SHBG levels were considerably lower in women with AD than in the BDs, one would expect the free fractions levels in AD to be increased relative to total hormone levels. However, previous reports indicate that SHBG levels in adrenal failure are in the middle-to-upper normal range, 65–90 nmol/l (34, 35, 36). Moreover, the postmenopausal women in our study were not on oral contraceptives. We, therefore, believe that the very low levels of total serum testosterone and androstenedione in women with AD indicate reduced bioactive androgens available to target tissues.

In conclusion, we developed a simple, automated, fast and highly specific LC–MS/MS assay that quantifies endogenous androgens and glucocorticoids, together with common synthetic glucocorticoids used in diagnosis and treatment. The method is highly sensitive and allows the measurement of androgens in women and children. The abilities of the method were illustrated by profiling steroid hormones in a large cohort of females with and without AD. The levels of androgens were lowest in women with AD across all age groups, but particularly in those aged above 60 years. This may suggest that androgen synthesis in the postmenopausal ovary depends on adrenal DHEA/S as the substrate.

## Supplementary data

This is linked to the online version of the paper at http://dx.doi.org/10.1530/EC-13-0023.

## Figures and Tables

**Table 1:** MRM transitions and compound-dependent parameters.

	**Quantifier**						**Qualifier**					
	MRM transition		Dwell time	DP	CE	CXP	MRM transition		Dwell time	DP	CE	CXP
**Period**	Q1	Q3	(ms)	(V)	(V)	(V)	Q1	Q3	(ms)	(V)	(V)	(V)
1												
Cortisol	407.2	297.0	35	−50	−42	−23	407.2	282.0	10	−50	−49	−23
Cortisol-d4	411.4	301.1	35	−50	−45	−23	411.4	286.1	10	−50	−48	−23
Prednisolone	405.2	295.0	35	−50	−42	−23	405.2	280.0	10	−50	−48	−23
Prednisolone-d6	411.3	333.2	35	−50	−23	−23	411.3	284.1	10	−50	−48	−23
Prednisone	403.3	299.1	35	−50	−25	−23	403.3	285.1	10	−50	−40	−23
Cortisone	405.3	301.1	35	−50	−26	−23	405.3	311.1	10	−50	−41	−23
Cortisone-d2	407.3	303.1	35	−50	−28	−23	407.3	313.0	10	−50	−41	−23
2												
DXM	437.4	361.1	80	−80	−24	−24	437.4	307.1	80	−80	−42	−15
DXM-d4	441.3	309.0	80	−80	−42	−20	441.3	363.2	80	−80	−24	−24
3												
11DOC	347.2	96.9	80	50	30	12	347.1	108.9	80	50	31	12
11DOC-d2	349.3	108.9	80	50	33	12	349.3	96.9	80	50	30	12
4												
Testosterone	289.2	97.0	80	85	30	12	289.2	109.0	80	85	33	12
Testosterone-d3	292.2	109.0	80	91	35	12	292.2	97.0	80	91	33	12
5												
Androstenedione	287.2	96.9	60	105	30	16	287.2	108.9	60	105	33	15
Androstenedione-d7	294.1	99.7	60	116	29	16	294.1	113.0	60	116	35	15
6												
17OHP	331.3	96.9	60	88	30	10	331.3	108.9	60	88	34	14
17OHP-d8	339.2	100.0	60	106	33	16	339.2	113.1	60	106	41	12
7												
Progesterone	315.3	96.9	35	95	30	12	315.3	108.9	35	95	28	12
Progesterone-d9	324.2	100.2	35	110	37	12	324.2	113.0	35	110	27	12

DP, declustering potential; CE, collision energy; CXP, collision exit potential; 11DOC, 11-deoxycortisol; DXM, dexamethasone; 17OHP, 17α-hydroxyprogesterone.

**Table 2:** Precision and accuracy.

	**Cortisol**	**Cortisone**	**Prednisolone**	**Prednisone**	**DXM**	**11DOC**	**Testosterone**	**Androstenedione**	**17OHP**	**Progesterone**
QC 1										
Mean	5.80	3.01	2.96	3.05	0.747	0.305	0.223	0.372	0.757	0.736
Within-day CV (%)	5.8	6.8	7.7	7.2	4.3	8.6	4.6	9.3	7.1	7.0
Total CV (%)	7.4	6.9	10.1	9.8	5.1	9.7	5.2	9.3	9.8	8.4
Accuracy (%)	97	100	99	102	100	102	99	99	101	98
QC 2										
Mean	101	50.4	51.3	53.8	12.5	5.00	3.71	6.10	12.2	12.5
Within-day CV (%)	3.0	3.9	4.3	3.8	3.7	4.7	5.1	3.9	4.5	6.1
Total CV (%)	4.5	4.7	5.3	8.7	4.8	5.7	5.6	5.0	7.7	6.9
Accuracy (%)	101	101	103	108	100	100	99	98	98	100
QC 3										
Mean	1004	–	517	535	128	–	36.4	61.6	121	118
Within-day CV (%)	4.7	–	5.0	4.7	4.0	–	4.7	4.1	4.6	4.5
Total CV (%)	5.7	–	5.5	8.8	5.2	–	4.9	5.0	8.0	6.8
Accuracy (%)	100	–	103	107	102	–	97	99	96	95

QCs were run in replicates of 6 on 12 different days. Total CV includes within-day and between-day variability. All concentrations are reported as nmol/l. QC 3 levels of cortisone and 11DOC are above the upper limit of quantification and thus omitted. CV, coefficient of correlation; 11DOC, 11-deoxycortisol; DXM, dexamethasone; 17OHP, 17α-hydroxyprogesterone.

**Table 3:** Limits of detection (LoDs) and lower limits of quantification (LLoQs).

	**Cortisol**	**Cortisone**	**Prednisolone**	**Prednisone**	**DXM**	**11DOC**	**Testosterone**	**Androstenedione**	**17OHP**	**Progesterone**
LoD (nmol/l)	<0.1	<0.1	<0.2	<0.2	<0.03	<0.03	<0.01	<0.02	<0.06	<0.06
LoQ (nmol/l)	1.95	1.58	0.49	0.97	0.061	0.098	0.018	0.122	0.244	0.122
CV (%)	11.4	6.7	12.3	18.3	5.4	17.3	18.8	16.9	14.2	16.9
Accuracy (%)	94.6	117.9	86.0	103.6	112.0	103.5	116.2	103.8	95.9	113.4

LoD is defined as a signal-to-noise ratio above 3. LoQ is defined as the lowest level that could be measured with accuracy within ±20% of the nominal levels and a CV below 20% (*n*=6). At the LLoQ, the signal-to-noise ratio was above 10 for all the compounds in all the replicates. DXM, dexamethasone; 11DOC, 11-deoxycortisol; 17OHP, 17α-hydroxyprogesterone.

**Table 4:** Steroid profiles of females with Addison's disease and in female blood donors.

	**Patients with Addison's disease**										**Blood donors**					
	Age below 40 years (*n*=25)		Age 40–59 years (*n*=71)		Age above 60 years (*n*=43)		POF (*n*=10)		DHEA (*n*=7)		Age below 40 years (*n*=48)		Age 40–59 years (*n*=41)		Age above 60 years (*n*=13)	
	<LoQ	Median (IQR)	<LoQ	Median (IQR)	<LoQ	Median (IQR)	<LoQ	Median (IQR)	<LoQ	Median (IQR)	<LoQ	Median (IQR)	<LoQ	Median (IQR)	<LoQ	Median (IQR)
Age (years)		35 (30–38)		48 (44–52)		68 (64–76)		56 (53–62)		51 (46–54)		28 (25–32)		48 (45–55)		66 (62–68)
Cortisol	0	326 (116–540)	2	273 (102–452)	0	290 (149–524)	0	404 (163–554)	1	133 (83.6–289)	0	290 (218–407)	0	211 (153–278)	0	256 (175–303)
Cortisone	0	58.5 (27.5–67.5)	2	45.1 (24.9–59.6)	1	51 (34.7–65.7)	0	53.5 (41.6–61.1)	1	40.6 (25.2–52.5)	0	44.8 (37.3–53)	0	41.5 (35.6–49.4)	0	42.1 (40.6–51.3)
Prednisolone	25	NA	69	0 (0–0)	41	0 (0–0)	9	0 (0–0)	7	NA	48	NA	41	NA	13	NA
Prednisone	25	NA	69	0 (0–0)	41	0 (0–0)	9	0 (0–0)	7	NA	48	NA	41	NA	13	NA
DXM	25	NA	71	NA	43	NA	10	NA	7	NA	48	NA	41	NA	13	NA
11DOC	22	0 (0–0)	66	0 (0–0)	42	0 (0–0)	10	NA	7	NA	0	0.30 (0.20–0.61)	1	0.29 (0.22–0.48)	0	0.32 (0.26–0.45)
Testosterone	2	0.44 (0.22–0.63)	13	0.26 (0.12–0.45)	16	0.15 (0–0.41)	5	0.03 (0–0.27)	0	0.56 (0.47–0.95)	0	0.71 (0.56–0.86)	0	0.56 (0.43–0.67)	0	0.65 (0.56–1.10)
Androstenedione	2	1.18 (0.71–1.76)	19	0.63 (0–10.2)	38	0 (0–0)	6	0 (0–0.16)	1	1.74 (0.87–1.85)	0	2.47 (1.87–2.81)	0	1.64 (1.18–2.21)	0	1.28 (0.96–1.64)
17OHP	5	0.65 (0.37–1.59)	26	0.41 (0–1.21)	40	0 (0–0)	8	0 (0–0)	4	0 (0–0.78)	6	0.68 (0.44–1.38)	7	0.68 (0.35–1.86)	3	0.31 (0.25–0.63)
Progesterone	16	0 (0–2.72)	43	0 (0–1.96)	43	NA	10	NA	5	0 (0–0.78)	23	0.249 (0–5.82)	23	0 (0–3.52)	13	NA

All levels are expressed as median (IRQ, interquartile range) nmol/l. <LoQ, number of samples below the limit of quantification; POF, premature ovarian failure; DHEA, DHEA replacement therapy; DXM, dexamethasone; 11DOC, 11-deoxycortisol; 17OHP, 17α-hydroxyprogesterone, NA, not applicable because hormone was not detected.
